# Generation of mixed murine organoids to model cellular interactions

**DOI:** 10.1016/j.xpro.2021.100997

**Published:** 2021-12-08

**Authors:** Ana Krotenberg Garcia, Jacco van Rheenen, Saskia Jacoba Elisabeth Suijkerbuijk

**Affiliations:** 1 Department of Molecular Pathology, Oncode Institute, Netherlands Cancer Institute, Amsterdam 1066 CX, The Netherlands

**Keywords:** Cancer, Cell Biology, Cell culture, Microscopy, Organoids

## Abstract

Cell competition is a mechanism of interaction that dictates cell selection based on differences in cellular fitness. We designed a protocol to generate mixed murine organoids and enteroid monolayers used to study such complex cellular interactions in a mammalian system. This protocol is dedicated to follow the behavior of different cell populations over time, using (time-lapse) microscopy or transcriptome/proteome analysis.

For complete details on the use and execution of this protocol, please refer to Krotenberg Garcia et al. (2021).

## Highlights

• Protocol to generate 3D mixed murine organoids and enteroid monolayers
• Analysis of complex cellular interactions, such as cell competition
• Follow the behavior of different cell populations over time
• Adaptable to other organoid cultures derived from different tissues

## Graphical abstract

**Figure:**
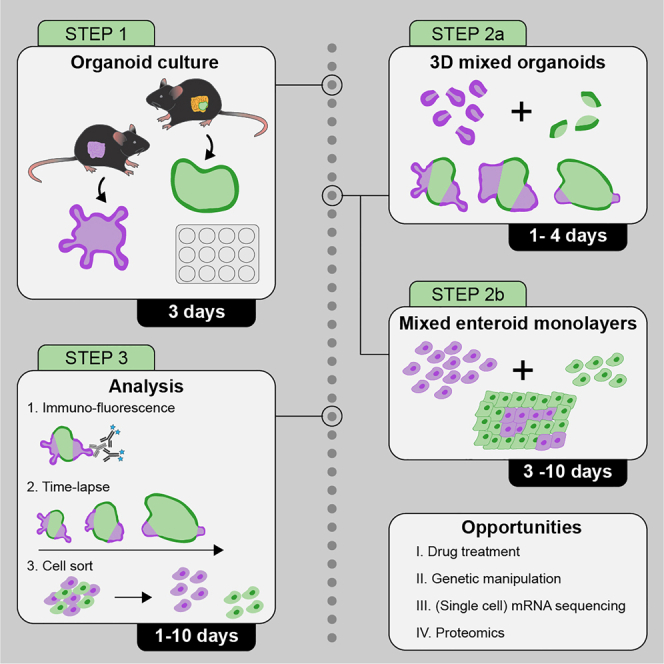

## Before you begin

In tissues, cellular interactions are essential for quality control, both during development and in homeostasis. In particular, competitive interactions can drive selection and thereby determine cell fate, growth and survival (Vishwakarma and Piddini, 2020). This mechanism was historically modeled in *Drosophila* (Simpson, 1979; Suijkerbuijk et al., 2016), Madin–Darby canine kidney (MDCK) epithelial cells (Hogan et al., 2009; Norman et al., 2012) and more recently inducible mouse models (Kon et al., 2017; Flanagan et al., 2021; Hill et al., 2021; van Neerven et al., 2021). Here, we describe a readily adaptable method to generate mixed organoid cultures, which mimic the close interaction between different cell populations.

This protocol describes the generation of mixed organoids and enteroid monolayers from two types of murine small intestine organoid cultures; wild-type cells (Krotenberg Garcia et al., 2021) and *Apc*^−/−^*Kras*^G12D/WT^*Trp53*^-/R172H^ cancer cells (Fumagalli et al., 2017). Previously we have extended the use of the protocol to other colorectal cancer lines (Jackstadt et al., 2019; Krotenberg Garcia et al., 2021). Based on the flexibility of the protocol it can be adapted to generate mixed organoids from other cystic-growing cultures derived from different tissues. The individual organoid cultures described in this protocol were derived from different donor mice with a similar genetic background (C57BL/6JRj). However, we have previously generated mixed organoids from cultures with a different genetic background and did not observe aberrant behavior.

For successful execution of this protocol and subsequent analysis, labeling of both cell populations is essential. In particular, when loss (out-competition) of one population is expected. Here we describe the use of wild-type intestine cells labeled with membrane-bound tdTomato (derived from mTmG transgenic mice (Muzumdar et al., 2007)) and cancer cells labeled with Dendra2 (introduced by lentiviral transduction (Heinz et al., 2020)).

Furthermore, the ratio of cells that are added to the mixing procedure is dependent on the characteristics of the original organoid cultures. For the intestinal model described here a 2:1 ratio (wild-type : cancer) is optimal. This ratio, which was based on the difference in proliferation rate between the individual cell populations and validated experimentally, provides equal competition potential to both populations. In addition, other characteristics such as cell ratios that naturally occur in organ tissue can serve as a guideline.

This protocol has broad applications and can be used to study cellular interactions on an organoid and population basis. Involvement of specific molecular pathways can be revealed through a combination with chemical or genetic manipulation or downstream analysis such as (single cell) mRNA sequencing or proteomics.

## Key resources table

**Table:** 

REAGENT or RESOURCE	SOURCE	IDENTIFIER
**Antibodies**		

anti-Phospho-c-Jun (Ser73) (D47G9) (Dilution 1:500)	Cell Signaling TECHNOLOGY	3270; RRID:AB_2129575
anti-Cleaved Caspase-3 (Asp175) (Dilution 1:400)	Cell Signaling TECHNOLOGY	9661; RRID:AB_2341188 06-570
anti-Aldolase B + Aldolase C antibody [EPR3138Y] (Dilution 1:300)	Abcam	ab75751; RRID:AB_2226682
anti-Lysozyme (EC 3.2.1.17) (Dilution 1:500)	Agilent	A0099; RRID:AB_2341230
anti-Olfm4 (D6Y5A) (1:100)	Cell Signaling TECHNOLOGY	39141; RRID:AB_2650511
Chicken anti-Rabbit, Alexa Fluor 647 (Dilution 1:500)	Thermo Fisher Scientific	A21443; RRID:AB_2535861

**Chemicals, peptides, and recombinant proteins**		

Cultrex PathClear Reduced Growth Factor Basement Membrane Extract Type 2	R&D Systems	3533-005-02
Advanced DMEM F/12	Thermo Fisher Scientific	12634-010
HEPES	Thermo Fisher Scientific	15630-056
Penicillin/streptomycin	Thermo Fisher Scientific	15140-122
GlutaMAX	Thermo Fisher Scientific	35050-068
R-spondin1	prepared in house	n/a
Noggin	prepared in house	n/a
B-27	Thermo Fisher Scientific	17504-044
hEGF	PeproTech	AF-100-15
N-acetylcysteine	Sigma-Aldrich	A9165
Y-27632	AbMole BioScience	M1817
CHIR-99021	Tocris Bioscience	4423
TryplE	Thermo Fisher Scientific	12605-010
DPBS, no calcium, no magnesium	Thermo Fisher Scientific	14190144
PBS tablets	Gibco	18912014
Paraformaldehyde 16% (w/v) in aqueous solution methanol-free	Alfa Aesar	43368.9M; CAS RN:50-00-0
BSA	Roche	10735094001
4′,6-Diamidino-2-phenylindole Dihydrochloride (DAPI)	Toronto Research Chemicals	D416050; CAS RN: 28718-90-3
TX-100	Sigma-Aldrich	X-100; CAS RN: 9036-19-5

**Deposited data**		

RNA-seq data	Krotenberg Garcia et al., 2021	NCBI GEO:GSE176027

**Experimental models: Cell lines**		

Murine WT small intestine organoids Gt(ROSA) 26Sortm4(ACTB-tdTomato,-EGFP)Luo/J Mus	Krotenberg Garcia et al., 2021	n/a
Murine CRC organoids Villin-CreERT2Apcfl/flKrasG12D/WTTr53fl/R1	Fumagalli et al., 2017	n/a

**Software and algorithms**		

FIJI	https://imagej.net/	2.1.0/1.53h 9.0.0
FlowJo 10.6.1	BD Biosciences	https://www.flowjo.com/
Imaris	Oxford Instruments	9.3.1

**Other**		

Cooled centrifuge	Eppendorf	Eppendorf Centrifuge 5702 R Serienr: 5703II619246
Eppendorf centrifuge	Eppendorf	Eppendorf AG 5424 Serienr: 5424FR273064
Tissue culture microscope equipped with fluorescent light source	Thermo Fisher Scientific	EVOS FL
Inverted confocal microscope	Leica Microsystems	TCS SP8
Spinning disk confocal microscope	Andor	Dragonfly
Cell counter	Westburg	Model: LUNA II Serienr: LUC-04-00126
Pasteur Capillary Pipette, short size 150mm	VWR	612-1798
5 mL Polystyrene Round-Bottom Tube with 35 μm Cell-Strainer Cap	Falcon	352235
5 mL Polystyrene Round-Bottom Tube	Falcon	352063
15 mL tubes	Sarstedt	62554502
1.5 mL Safe-Lock Tubes	Eppendorf	0030120.086
24 well CELLSTAR® plate, polystyrene	Greiner Bio-One	662165
12 well CELLSTAR® plate, polystyrene	Greiner Bio-One	665165
6 well CELLSTAR® plate, polystyrene	Greiner Bio-One	657165
8 well chambered μ-Slide, glass-bottom	IBIDI	80827
96 well μ-Plate, ibiTreat #1.5 polymer coverslip	IBIDI	89626
96 well SensoPlate, glass-bottom	Greiner Bio-One	655892
384 well microplate, tissue culture treated	Greiner Bio-One	781091

## Materials and equipment

### Culture media recipes

According to Sato et al. (2009) and Thorne et al. (2018)

**Table:** Basic culture medium

Reagent	Stock concentration	Final concentration	Amount
Advanced DMEM/F-12	n/a	n/a	500 mL
HEPES	1 M	10 mM	5 mL
GlutaMAX	100×	1%	5 mL
Penicillin/Streptomycin	10.000 U/mL	100 U/mL	5 mL
**Total**			**515 mL**

Prepare in a sterile environment, store at 4°C for up to 2 months.

**Table:** ENR culture medium (ENR: EGF, Noggin and RSPO1)

Reagent	Stock concentration	Final concentration	Amount
Basic culture medium	n/a	n/a	38.87 mL
B-27	50×	2%	1 mL
N-Acetyl-L-Cysteine	500 mM	1.25 mM	125 μL
R-spondin1 Conditioned Medium (CM)	n/a	10%	5 mL
Noggin Conditioned Medium (CM)	n/a	10%	5 mL
human Epidermal Growth Factor (hEGF)	0.5 mg/mL	50 ng/mL	5 μL
**Total**			**50 mL**

Prepare in a sterile environment, store at 4°C for up to 2 weeks.

***Note:*** For in house production of conditioned media refer to Broutier et al. (2016). Alternatively, Noggin-FC fusion protein and R-Spondin 3-FC fusion protein conditioned medium can be used (U-Protein Express BV, Cat. No.#N002 and #R001).

**Table:** Enteroid plating medium

Reagent	Stock concentration	Final concentration	Amount
Basic culture medium	n/a	n/a	38.77 mL
B-27	50×	2%	1 mL
N-Acetyl-L-Cysteine	500 mM	1.25 mM	125 μL
R-spondin1 CM	n/a	10%	5 mL
Noggin CM	n/a	10%	5 mL
hEGF	0.5 mg/mL	50 ng/mL	5 μL
CHIR-99021	3 mM	3 μM	50 μL
Y-27632	10 mM	10 μM	50 μL
**Total**			**50 mL**

Prepare in a sterile environment, store at 4°C for up to 2 weeks.

### Solution recipes

**Table:** Fixation solution

Reagent	Stock concentration	Final concentration	Amount
Paraformaldehyde (methanol-free)	16%	4%	10 mL
PBS	2× from tablets	1×	20 mL
MilliQ water	n/a	n/a	10 mL
**Total**			**40 mL**

Store at -20°C for up to 1 month, avoid freeze thaw cycles.

**CRITICAL:** Paraformaldehyde is hazardous; wear gloves, work in chemical safety hood and dispose waste in accordance with local regulations.

**Table:** Blocking solution

Reagent	Stock concentration	Final concentration	Amount
BSA	n/a	5%	2.5 g
TX-100	10%	0.2%	1 mL
PBS	1× from tablets	1×	49 mL
**Total**			**50 mL**

Store at 4°C for up to 1 month.

**Table:** Antibody incubation solution

Reagent	Stock concentration	Final concentration	Amount
BSA	n/a	2.5%	1.25 g
TX-100	10%	0.1%	0.5 mL
PBS	1× from tablets	1×	49.5 mL
**Total**			**50. mL**

Store at 4°C for up to 1 month.

**Table:** Washing solution

Reagent	Stock concentration	Final concentration	Amount
TX-100	10%	0.1%	0.5 mL
PBS	1× from tablets	1×	49.5 mL
**Total**			**50 mL**

Store at room temperature (18°C–25°C) for up to 6 months.

**Table:** FACS buffer

Reagent	Stock concentration	Final concentration	Amount
dPBS	n/a	n/a	9.75 mL
B-27	50×	2%	200 μL
N-Acetyl-L-Cysteine	500 mM	1.25 mM	25 μL
hEGF	0.5 mg/mL	50 ng/mL	1 μL
Y-27632	10 mM	10 μM	10 μL
DAPI	1 mg/mL	1 μg/mL	10 μL
**Total**			**10 mL**

Prepare in a sterile environment, store at 4°C for up to 2 weeks.

### Media and reagent preparation

**Timing: 2 days**

**Figure 1:**
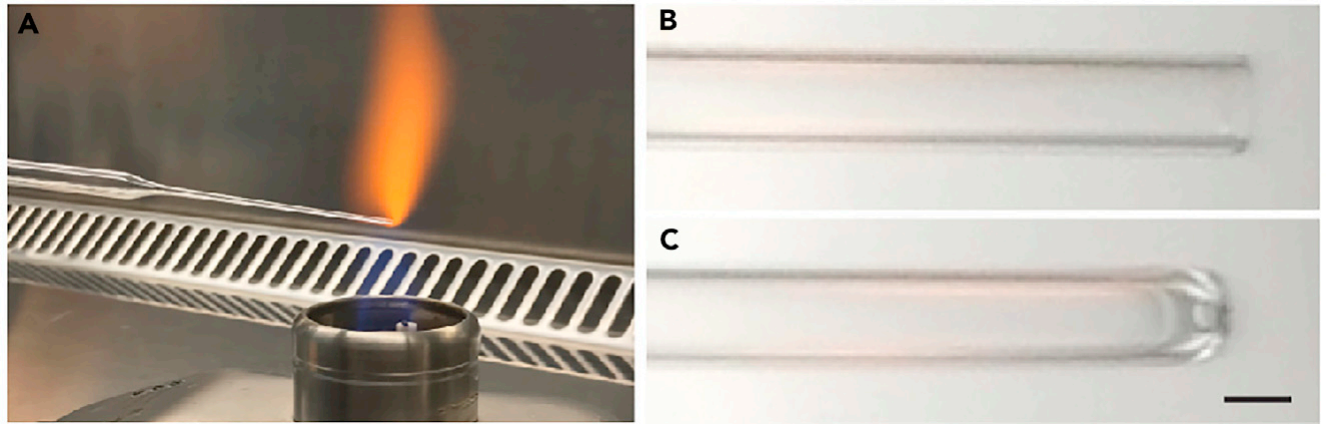
Preparation of glass Pasteur pipets (A) Fire-polish a glass Pasteur pipet by placing the tip in the flame of a Bunsen burner for a couple of seconds while continuously rolling the pipet. (B and C) An example of a glass Pasteur pipet before (B) and after (C) fire-polishing. Scale bar = 1 mm
• Cool centrifuges to 4°C.

***Note:*** Instead of BME2 other culture matrices (e.g. Matrigel or Collagen) can be used. Select appropriate matrix depending on organoid culture of choice. Specific characteristics of certain matrices might interfere with downstream analysis. For example, Collagen does not dissolve well in the fixation solution (Step 33) and might need additional permeabilization steps, or Matrigel containing phenol-red might cause problems with auto-fluorescence in time-lapse experiments. Optimization is recommended when alternative matrices are used.

• Thaw Reduced Growth Factor Basement Membrane Extract Type 2 (BME2) on ice or in a fridge overnight.
• Incubate unpackaged 6, 12 and 24 well plates (depending on the type of experiment) untreated polystyrene culture plates in a tissue culture incubator at 37°C for at least 48h prior to use to promote formation of BME2 droplets during plating.
• Prepare all buffers and media according to the tables in the “Materials and equipment” section and store appropriately (see notes).
• Prepare glass Pasteur pipets by fire-polishing the tip of the pipets using a Bunsen burner (Figure 1). At least one pipet per individual culture should be prepared, spares are recommended.

## Step-by-step method details

### Organoid culture

**Timing: 7 days**

**Figure 2:**
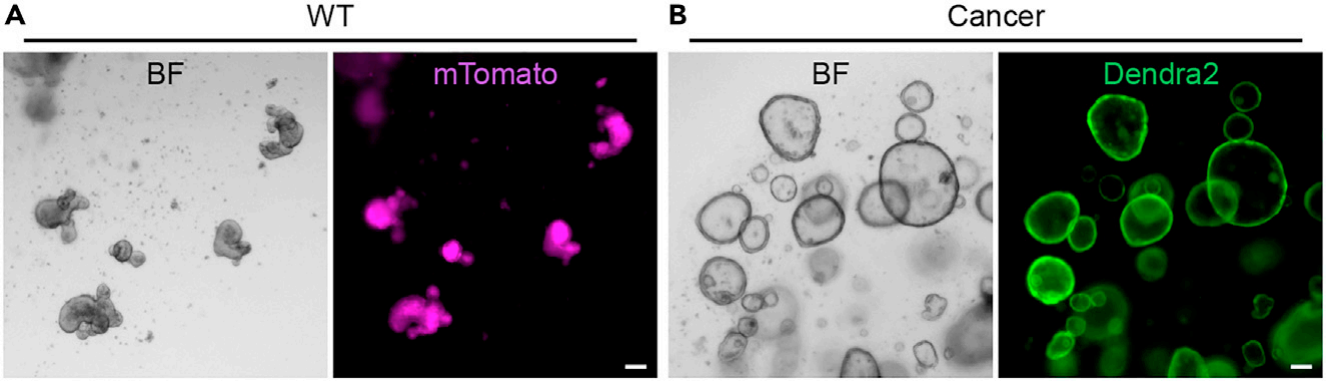
Input organoid cultures (A and B) Mature murine wild-type (A) and Cancer (B) organoid cultures passaged 3 days prior to the start of the experiment. Pay attention to the morphology of the organoids, which should have a smooth epithelium and lack apoptotic cells in their lumen. 2–6 crypt-like structures are optimal for wild-type small intestine cultures. (A) Membrane-bound tdTomato labelled wild-type cells (Krotenberg Garcia et al., 2021) were derived from healthy small intestine tissue. (B) Dendra2 labelled cancer cells were derived from *Apc*^−/−^*Kras*^G12D/WT^*Trp53*^-/R172H^ small intestinal tumors (Fumagalli et al., 2017). Scale bars = 100 μm

This section describes the culture steps in preparation of small intestine wild-type and cancer organoids for generation of mixed cultures. For complete details on the isolation and culture of murine small intestine organoids please refer to (Sato et al., 2009; Sato and Clevers, 2012; Andersson-Rolf et al., 2014). For optimal and efficient execution of this protocol it is essential that the organoids have adapted to the experimental culture medium and are in a healthy state (Figure 2) prior to the start of the experiment.

1. Culture the organoids in 3D in BME2 for at least 7 days in ENR culture medium. There is no maximum duration of this adaptation time. Passage the cultures when the lumen fills with apoptotic cells, approximately once every 3–4 days.

**CRITICAL:** It is essential that the experimental culture medium is uniform for all organoid cultures in the experiment and contains a combination of all factors that are required for the individual cultures. For example, the cancer population described in this protocol does not require hEGF or RSPO1, but is cultured in ENR medium, in order to avoid phenotypic changes during the experiment that could be caused by growth factors.

2. Passage organoid cultures 2–3 days prior to the start of the experiment until mature and healthy organoids are formed (Figure 2). Refer to Table 2 for guidance on input material.

***Note:*** We have not observed differences in outcome between early and late passage cultures. However, depending on the organoid cultures of choice the use of early passages might be required.

### Preparation of organoids

**Timing: 15–30 min**

This section describes the preparation of small intestine wild-type and cancer organoids for mixed cultures. After Step 7, organoids are ready to proceed to generation of 3D mixed organoids (Part A) or generation of mixed enteroid monolayers (Part B).

***Note:*** Organoid cultures are very sensitive to fluctuations in temperature. Therefore, we recommend to use ice-cold reagents, keep cells on ice in between steps and use centrifuges that are cooled to 4°C throughout the protocol.

3. Harvest organoids by scraping the bottom of the well and pipetting up and down with a p1000 pipette using ice-cold Basic culture medium.
4. Collect the organoid suspension in 15 mL centrifuge tubes and keep on ice.

***Optional:*** To ensure harvest of all organoids, it is recommended to wash the well with another 1 mL of ice-cold Basic medium and collect leftovers.

5. Centrifuge at 300×*g* at 4°C for 5 min. Three clear layers should be formed; medium, BME2 and the pellet of organoids (top-to-bottom, Figure 3).

**Figure 3:**
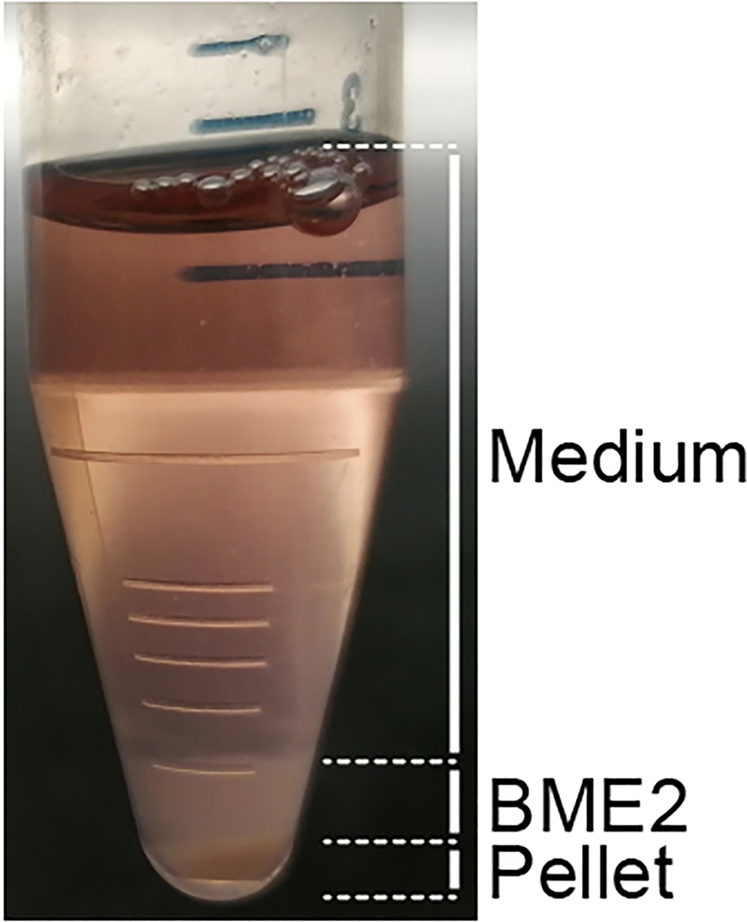
Preparation of organoids Overview of the three phases formed by centrifugation of an organoid suspension. Medium (top), BME2 (middle) and organoid pellet (bottom).
6. Carefully remove the medium and BME2 layers without disrupting the organoid pellet.
7. Resuspend the pellet in 1 mL of ice-cold Basic culture medium.

### Part A. Generation of 3D mixed organoids

**Timing: 2–3 h**

This section describes the main steps to generate 3D mixed organoids from small intestine wild-type and cancer cells. Once the 3D mixed organoids are formed, they can be used in different techniques such as cell sorting, immuno-fluorescence and time-lapse imaging, described later in this protocol.

**Figure 4:**
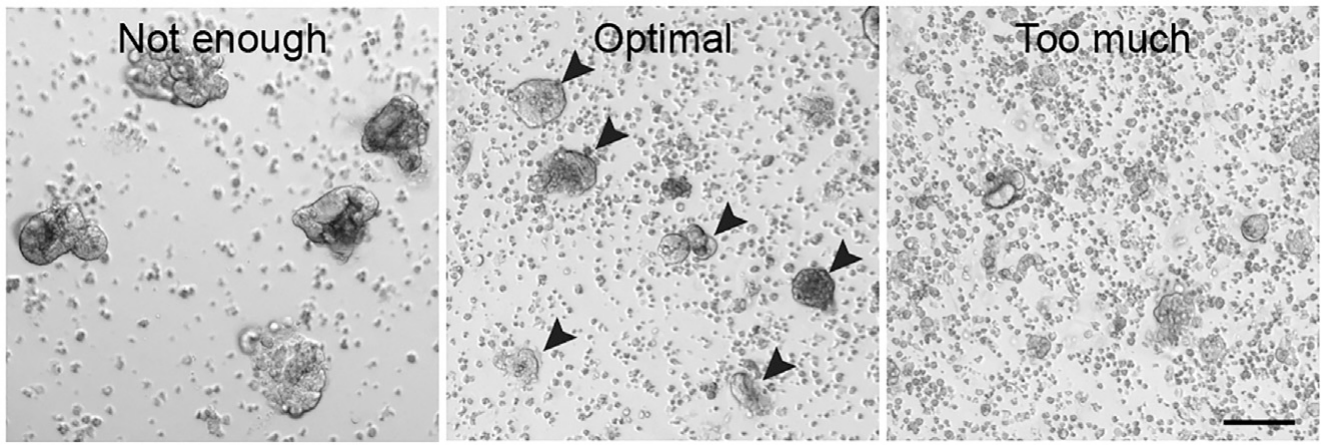
Generation of organoid clump suspension Bright field examples of mechanically disrupted organoid cultures. Depicted are fragments that need more disruption (left), of optimal size (middle, indicated by arrow heads) or were disrupted too much (right). Scale bar = 100 μm

***Note:*** Immediately before use, sterilize glass Pasteur pipets by briefly passing them through a flame and coat the inside walls of the pipet by aspiration of Basic culture medium to avoid attachment of the organoids.

8. Use a fire-polished glass Pasteur pipet to mechanically disrupt the organoids. Pipette up and down 10–20× while pressing the pipet tip against the bottom of the tube, until no clear structures are visible by eye. The suspension now contains clumps of 50–150 cells (Figure 4).

**CRITICAL:** Optimal breaking of the organoids is essential. Therefore, it is recommended to monitor the status at a microscope after every 5–10× of pipetting. Stop the breaking of the organoids before they are single cells (Figure 4).

9. Centrifuge at 300×*g* at 4°C for 5 min.
10. Aspirate supernatant and resuspend the pellets in 300 μL ice-cold Basic culture medium.

**CRITICAL:** The final ratio of cells in the mixed organoids is dependent on the input of the different cell populations (see Table 2). Therefore, it is important to compare the pellet size and adjust the amount of Basic culture medium that is added in Step 10 accordingly (add 300 μL to the smallest pellet, increase volume X times for a X times bigger pellet).

11. Prepare one Eppendorf tube for each of the conditions (Pure population A, Pure population B and Mix Population A : B) and add the correct amount of cell clump suspension (Table 1).

**Table 1:** Recommended volumes of cell clump suspension for mixing of 3D organoids

Condition	Ratio	Pure population A	Pure population B	Mix population A : B
1	1:1	**Vial 1A:** 100 μL	**Vial 1B:** 100 μL	**Vial 1AB:** 100 μL : 100 μL
2	2:1	**Vial 2A:** 100 μL	**Vial 2B:** 50 μL	**Vial 2AB:** 100 μL : 50 μL
3	3:1	**Vial 3A:** 100 μL	**Vial 3B:** 33 μL	**Vial 3AB:** 100 μL : 33 μL
4	4:1	**Vial 4A:** 100 μL	**Vial 4B:** 25 μL	**Vial 4AB:** 100 μL : 25 μL

***Note:*** The efficiency of generating mixed organoids is not 100%. Therefore, it is recommended to include duplicates of mixed conditions in order to generate equal numbers of pure and mixed organoids.

***Note:*** The optimal ratio for mixing is dependent on the characteristics of the individual cultures, such as differences proliferation rate. It is recommended to include multiple ratios and validate the optimal mix conditions experimentally.

12. Centrifuge at 300×*g* at 4°C for 5 min
13. Carefully aspirate the supernatant with a p200 pipette and resuspend the small pellet in 10 μL of ENR medium.
14. Incubate the Eppendorf vials containing the concentrated cell clump suspensions at 37°C for 30 min to promote cell aggregation.

**CRITICAL:** Aggregation of cells in a small volume is essential for efficient formation of mixed organoids. Do not increase the volume to more than 25 μL.

15. After the incubation time, dilute the aggregates with ENR medium (see Table 2 for volume).

**Table 2:** Recommended input and plating conditions for mixing of 3D organoids

Input per well for four conditions			Aggregation per condition			Plating per condition	
Plate type	Size	No. organoids	Volume	+ENR medium	+BME2	No. drops	Drop size
24-well (Greiner)	1.9 cm^2^	250	5 μL	10 μL	30 μL	3	12 μL
12-well (Greiner)	3.9 cm^2^	500	10 μL	15 μL	50 μL	6	12 μL
6-well (Greiner)	9.6 cm^2^	1000–1500	20 μL	50 μL	140 μL	15	12 μL
16. Add double the volume of BME2 (compared to the diluted aggregates) to achieve a final ratio of 1:2 (see Table 2 for volume) and keep on ice.
17. Plate drops of 12 μL per well of an imaging-compatible plate. Output and drop size are based on use of a 96-well imaging plate (IBIDI). Adapt when different plates are used.

***Note:*** For sorting of cells from 3D organoids it is recommended to plate mixed organoids in a 6 well format to increase yield

***Note:*** The input material is indicated per one well, this is sufficient for four conditions (1× each of pure populations and 2× mix)

***Note:*** The volumes in Table 2 are guidelines. To avoid crowding it is recommended to plate one drop in a separate dish and monitor organoid density (Figure 5). Adjust the volume if needed.

***Note:*** The indicated drop size is optimized for plating of a single drop per well of a 96-well imaging plate (IBIDI). It is recommended to adjust the size when other plate types are used.

18. Invert the plate and let the drops solidify in a 37°C incubator for 10–30 min.

**Figure 5:**
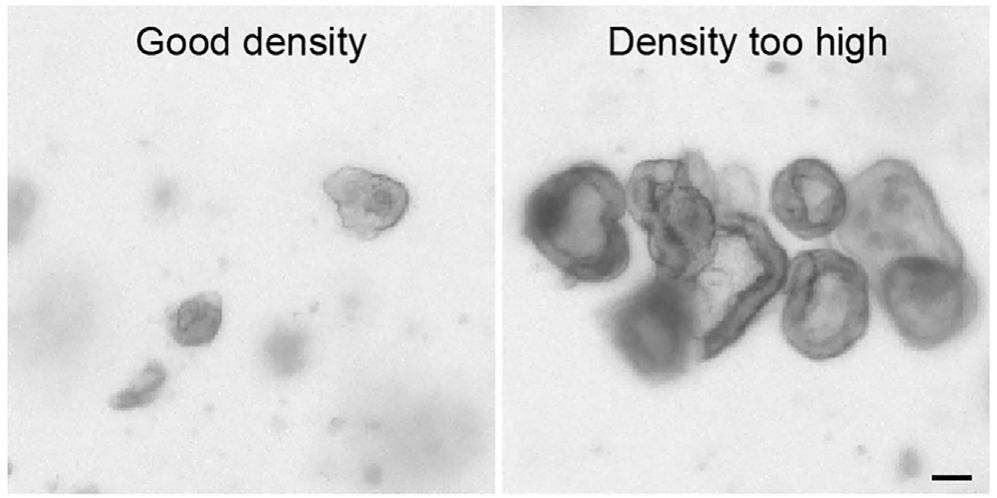
Plating density Bright field examples of cell aggregates that are plated at an optimal density (left) or a density that is too concentrated (right). Scale bar = 100 μm
19. Carefully add 150 μL room temperature ENR medium per well, do not disrupt the drops, and return the plate to the incubator (37°C, 5% CO2).

***Note:*** At this stage chemical manipulation can be started, for example by addition of small molecule inhibitors to the culture medium.

### Part B. Generation of mixed enteroid monolayers

**Timing: 2–3 h**

This section describes the main steps to generate mixed enteroid monolayers (Thorne et al., 2018) from individual small intestine wild-type and cancer cells. Once the monolayers are formed, they can be used in different techniques such as immuno-fluorescence and time-lapse imaging, described later in this protocol.

20. Coat imaging plates:
  a. Dilute BME2 to a final concentration of 0.8 mg/mL in ice-cold Basic culture medium and keep on ice.
  b. Add 50–150 μL of diluted BME2 per well (see Table 3 for appropriate volume) and incubate for 1 h at 37°C

**Table 3:** Recommended input and plating conditions for mixed enteroid monolayers

Well format			Number of cells /well					
			Ratio 1:1		Ratio 1:2		Ratio 1:4	
Type	Size	Volume	A	B	A	B	A	B
384 (Greiner)	0.10 cm^2^	50 μL	3000	3000	3000	1500	3000	750
96 (Greiner)	0.34 cm^2^	100 μL	10000	10000	10000	5000	10000	2500
96 (IBIDI)	0.56 cm^2^	150 μL	15000	15000	15000	7500	15000	3750
  c. Wash the coated wells twice with Basic culture medium and use the plate within 2 h. Keep the plates at room temperature until use.
21. Continue with resuspended pellet (Step 7) and use a fire-polished glass Pasteur pipet to mechanically disrupt the organoids by briefly pipetting up and down.

***Note:*** Immediately before use, sterilize glass Pasteur pipets by briefly passing them through a flame and coat the inside walls of the pipet by aspiration of Basic culture medium to avoid attachment of the organoids.

22. Centrifuge at 300×*g* at 4°C for 5 min.
23. Aspirate the supernatant and add 1 mL of TrypLE at room temperature.
24. Dissociate the broken organoids by pipetting up and down using a fire-polished glass Pasteur pipet.
25. Once a single cell suspension is formed (after ± 3 min at room temperature) immediately add 10 mL of cold Basic culture medium and keep on ice.

***Note:*** TrypLE is sufficiently inactivated by dilution. Additional inactivation steps (e.g. addition of serum or an inhibitor) might be required when other dissociation reagents are used. Check recommendations of the supplier.

**CRITICAL:** Some organoid cultures are very sensitive to dissociation. It is recommended to reduce the incubation time in TrypLE to less than 5 min. This can be achieved by processing few samples simultaneously and repeated monitoring at a microscope until a single cell suspension is reached (similar to the right panel in Figure 4).

26. Centrifuge at 300×*g* at 4°C for 5 min.
27. Aspirate the supernatant and add 200 μL of ice-cold Enteroid plating medium.
28. Count cells using a Bürker Türk Counting Chamber or automated cell counter
29. Dilute and plate cells in ice-cold Enteroid plating medium in an imaging compatible plate according to Table 3.
30. Let the cells adhere in an incubator (37°C, 5% CO_2_).
31. Replace the medium with 37°C ENR medium after 16–24 h and refresh every 2–3 days.

***Note:*** At this stage chemical manipulation can be started, for example by addition of small molecule inhibitors to the culture medium.

### Immuno-fluorescence staining

**Timing: 2 days**

This section describes how to proceed with Immuno-fluorescence staining of mixed organoids or enteroid monolayers. The indicated volumes are based on use of a 96-well imaging plate (IBIDI) and can be adapted accordingly when different plates are used.

32. Carefully aspirate culture medium without disturbing the drop of BME2 or the monolayer.
33. Add 100 μL Fixation solution (see “materials and equipment” section) and incubate at room temperature for 20 min.

***Note:*** It is not required to include an additional wash step prior to fixation.

**Figure 6:**
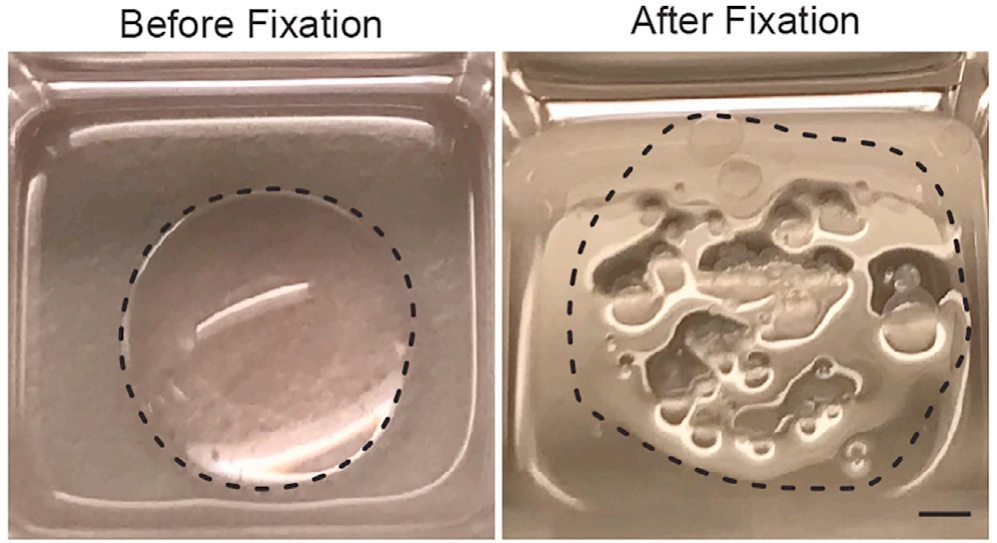
Fixation of mixed organoids Drop of BME2 containing mixed 3D organoids before fixation (left) and after dissolving in fixation solution (right). The BME2 drop, before and after disruption, are outlined by a dashed line. Scale bar = 1 mm

***Note:*** If the drop is not completely dissolved, the fixation can be prolonged for up to 30 min.

***Note:*** The organoids are now attached to the bottom of the well.

34. Aspirate the Fixation solution once the drop of BME2 is completely dissolved (Figure 6) and wash twice with 100 μL PBS.

**Pause point:** After fixation organoids can be stored in PBS for 2/3 weeks, protected from light at 4°C before continuing with immuno-staining.

35. Add 100 μL blocking solution (see “materials and equipment” section) and incubate at room temperature for at least 30 min to block and permeabilize organoids.

***Note:*** This step can be prolonged for several hours when convenient.

36. Add the appropriate primary antibodies diluted in Antibody incubation solution (50 μL per well) and incubate at 4°C protected from light overnight (8–16 h) or for up to several days.

***Note:*** See “expected outcomes” section for examples of antibodies that can be used.

37. Wash three times for 5 min with 100 μL Washing solution at room temperature.
38. Add the appropriate secondary antibodies diluted in Antibody incubation solution (50 μL per well) and incubate at room temperature protected from light for at least one hour.

***Note:*** At this step other dyes such as DAPI or phalloidin can be included. Selection of secondary antibodies should be based on the expression of fluorophores by the organoid cultures to avoid cross-talk.

***Note:*** This step can be prolonged for up to several days. Incubate at 4°C when exceeding 5 hours.

39. Wash three times with 100 μL Washing solution for 5 min each at room temperature.
40. Rinse twice with 100 μL PBS and leave the organoids in PBS.

**Pause point:** Stained organoids can be can be stored in the dark at 4°C. To prevent loss of signal, we recommend proceeding with image analysis within one week. Ensure the organoids do not dry out by adding enough PBS and/or sealing plates with parafilm.

41. Proceed to imaging using a platform of choice. A confocal based platform is recommended, using a high NA 20–25× objective.
42. Acquire Z-stacks, see notes for recommendations.

***Note:*** For 3D mixed organoids the thickness of Z-slices should be determined based on the detail of information that is needed for further analysis. For example, a thickness of 5 μm is sufficient for analysis at a cellular resolution. For optimal 3D reconstruction without loss of data, a system calculated optical slice thickness can be used.

***Note:*** For enteroid monolayers, it is recommended to acquire Z-stacks covering the total thickness of the monolayer (approximately 20 μm) to avoid loss of 3D information. Data can be further analyzed via 3D reconstruction or Z-projection.

### Time-lapse imaging of mixed organoids

**Timing: 1–4 days**

This section describes how to proceed with time-lapse imaging of mixed organoids or enteroid monolayers. Ensure cells are plated in an imaging-compatible plate at Step 17 or 29.

43. Refresh the medium 1 day after plating.
44. Add PBS to the surrounding empty wells to avoid evaporation.
45. Proceed to imaging using a platform of choice that is equipped with a CO_2_ and 37°C incubator. A (spinning-disk) confocal based platform is recommended, using a high NA 20–25× objective.
46. Acquire images with a maximum time interval of one hour and a maximum Z-slice thickness of 5 μm for tracking of single cells in 3D.

***Note:*** Organoids can be imaged for up to 72 hours. A change of medium is necessary for longer experiments.

### Sorting cells from mixed organoids

**Timing: 2–4 h**

This section describes how to process previously mixed 3D organoids for cell sorting.

47. Harvest organoids by scraping the bottom of the well and pipetting up and down with a p1000 pipette using ice-cold Basic culture medium.
48. Collect the organoid suspension in 15 mL centrifuge tubes.
49. Centrifuge at 300×*g* at 4°C for 5 min. Three clear layers should be formed; medium, BME2 and the pellet of organoids (top-to-bottom, Figure 3).
50. Carefully remove the medium and BME2 layers without disrupting the organoid pellet.
51. Resuspend the pellet in 1 mL of ice-cold Basic culture medium.
52. Mechanically disrupt the organoids by briefly pipetting up and down using a fire-polished glass Pasteur pipet.
53. Centrifuge at 300×*g* at 4°C for 5 min.
54. Aspirate the supernatant and add 1 mL of TrypLE at room temperature.
55. Dissociate the broken organoids by pipetting up and down using a fire-polished glass Pasteur pipet.
56. Once a single cell suspension is formed (after ± 3 min) immediately add 10 mL of ice-cold Basic culture medium and keep on ice.

***Note:*** TrypLE is sufficiently inactivated by dilution. Additional inactivation steps (e.g. addition of serum or an inhibitor) might be required when other dissociation reagents are used. Check recommendations of the supplier.

57. Centrifuge at 300×*g* at 4°C for 5 min.
58. Resuspend pellet in 250 μL ice-cold FACS buffer and pass through a 35 μm cell strainer.
59. Keep cells on ice and immediately proceed to cell sorting using a platform of choice.

## Expected outcomes

**Figure 7:**
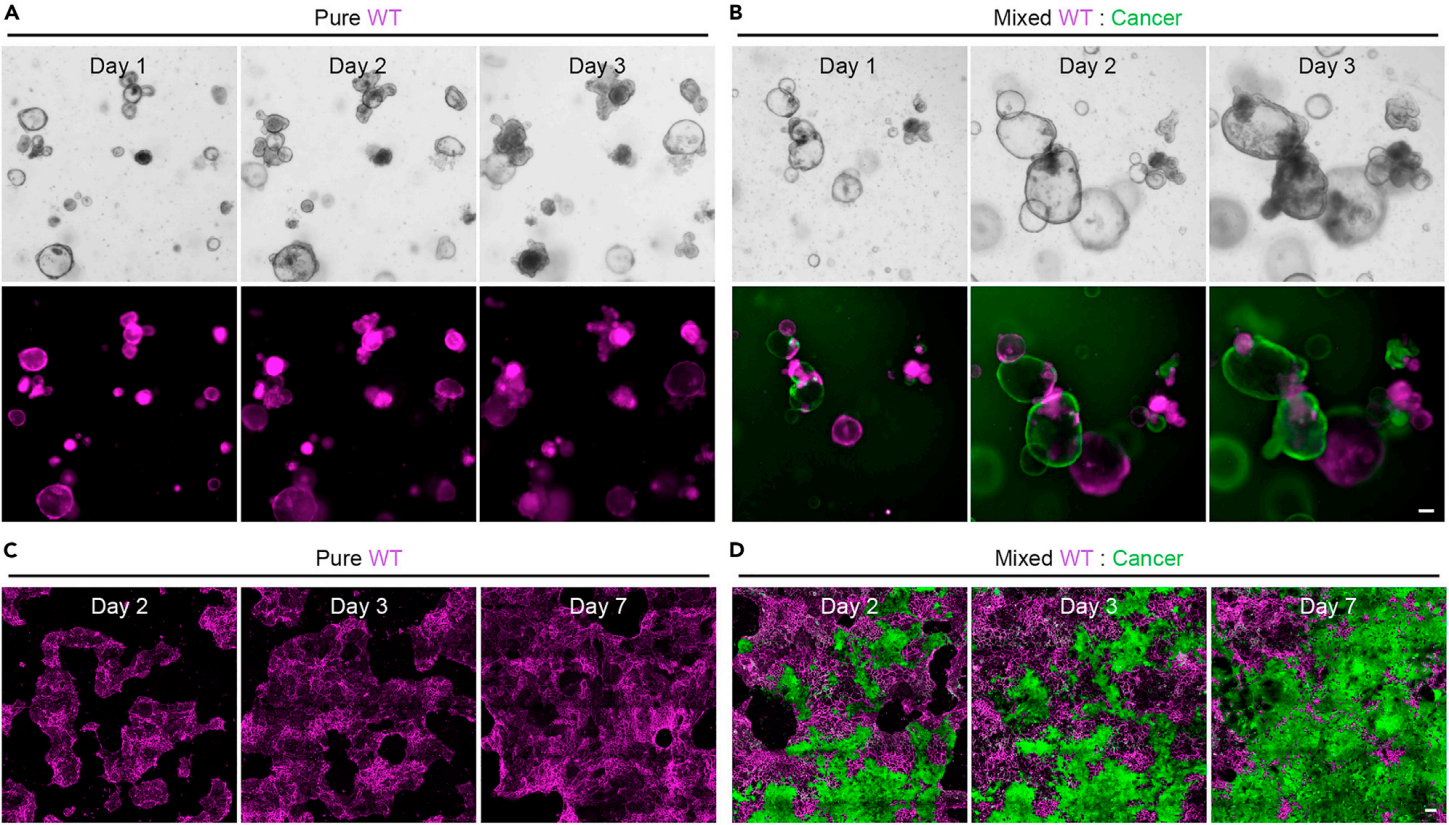
Progression of mixed 3D organoids and enteroid monolayers (A–D) Monitoring growth of pure (A) and mixed (B) 3D organoids using a fluorescent tissue culture microscope and pure (C) and mixed (D) enteroid monolayers using a stage-calibrated confocal microscope for up to one week. Cancer cells are visualized in green. The wild-type population (magenta) is gradually lost from the cultures over time. Scale bars = 100 μm

Our protocol describes the generation of mixed 3D organoids and enteroid monolayers from individual cell populations. We have not tried to derived mixed enteroid monolayers directly from mixed 3D organoids. During the course of the experiment growth and progression of the cultures can be monitored using a tissue culture microscope equipped with fluorescent light source. When generating mixed cultures of populations of different cellular fitness, a gradual loss of the weaker cell population is expected (Figure 7). The 3D cultures of wild-type small intestine and *Apc*^−/−^*Kras*^G12D/WT^*Trp53*^-/R172H^ cancer cells mixed in a 2:1 ratio described here, consist of ± 40% wild-type cells 1 day after mixing and this decreases to ±10% on day 4 (Krotenberg Garcia et al., 2021). The described enteroid monolayers mixed in a 2:1 ratio (wild-type : cancer) are composed of ±20% wild-type cells the moment a confluent monolayer has formed (Krotenberg Garcia et al., 2021).

**Figure 8:**
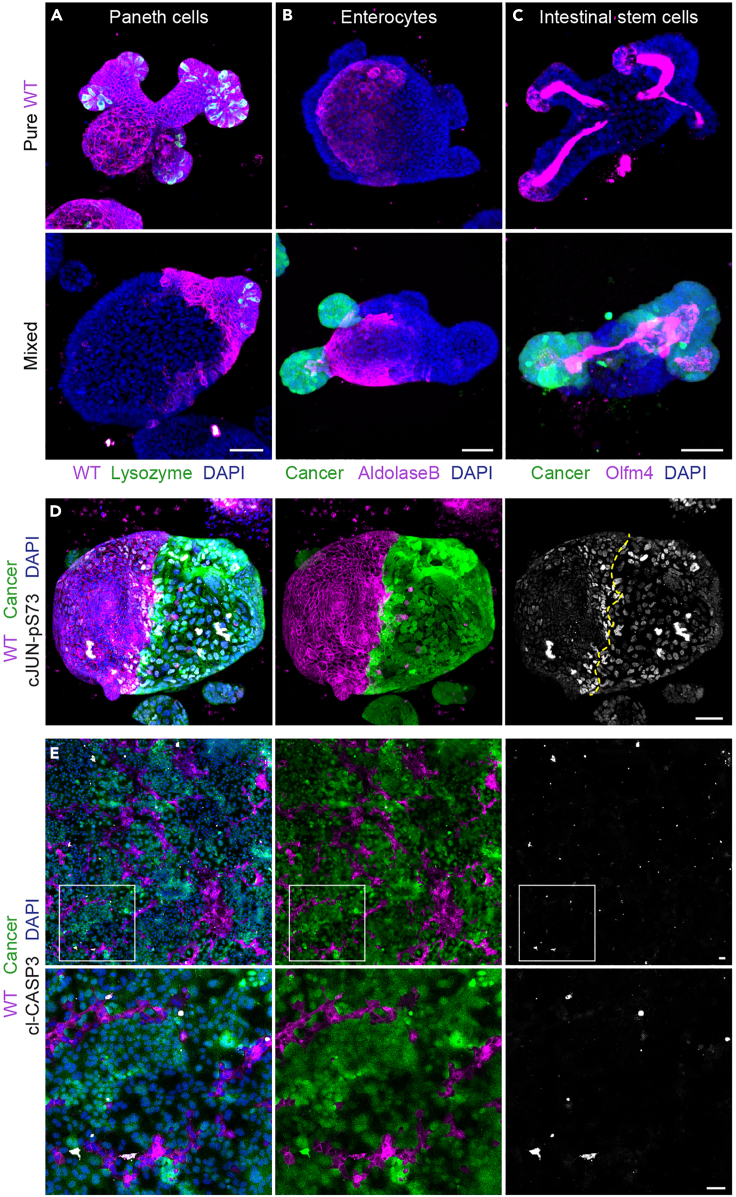
Analysis of cellular interactions using immuno-fluorescence Examples of analysis of interactions between cells in mixed 3D organoids and enteroid monolayers by immuno-fluorescence. (A–C) Immuno-fluorescence staining of 3D-reconstructed pure (top) and mixed (bottom) organoids. Cancer (green) or wild-type (magenta) cells are visualized. The organoids were stained for lysozyme (A, green, Paneth cells), Aldolase B (B, magenta, enterocytes) or OLFM4 (C, magenta, intestinal stem cells), nuclei are visualized with DAPI (blue). (D and E) Immuno-fluorescence staining of 3D-reconstructed mixed organoid (D) and mixed monolayer (E). Cancer (green) and wild-type (magenta) cells are visualized. The organoids were stained for cJUN-pS73 (D, grey) or cl-CASP3 (E, grey); nuclei are visualized with DAPI (blue). Also see Methods video S1. Scale bars = 50 μm

**Figure 9:**
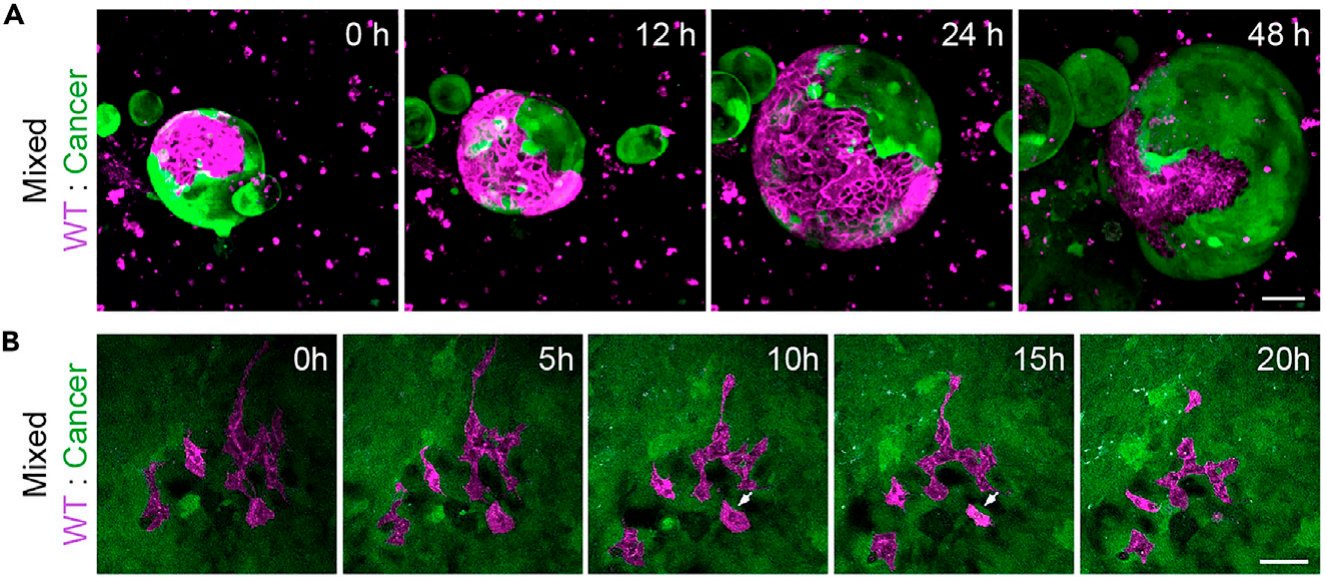
Analysis of cellular interactions using time-lapse microscopy (A) Time-lapse series of a 3D-reconstructed mixed organoid, started one day after mixing. Cancer (green) and wild-type (magenta) cells are visualized. Also see Methods video S2.Time-lapse series of control treated 3D reconstructed mixed intestinal organoid, related to steps 43–46. (B) Time-lapse series of a mixed monolayer. Image acquisition was started after a full monolayer had formed, approximately three days after plating and the ratio shifted towards the cancer population. Cancer (green) and wild-type (magenta) cells are visualized. The arrow indicates an eliminated WT cell, which shrinks and is lost from the culture. Also see Methods video S3.Time-lapse series of a competing enteroid monolayer, related to steps 43–46. Scale bars = 50 μm

**Figure 10:**
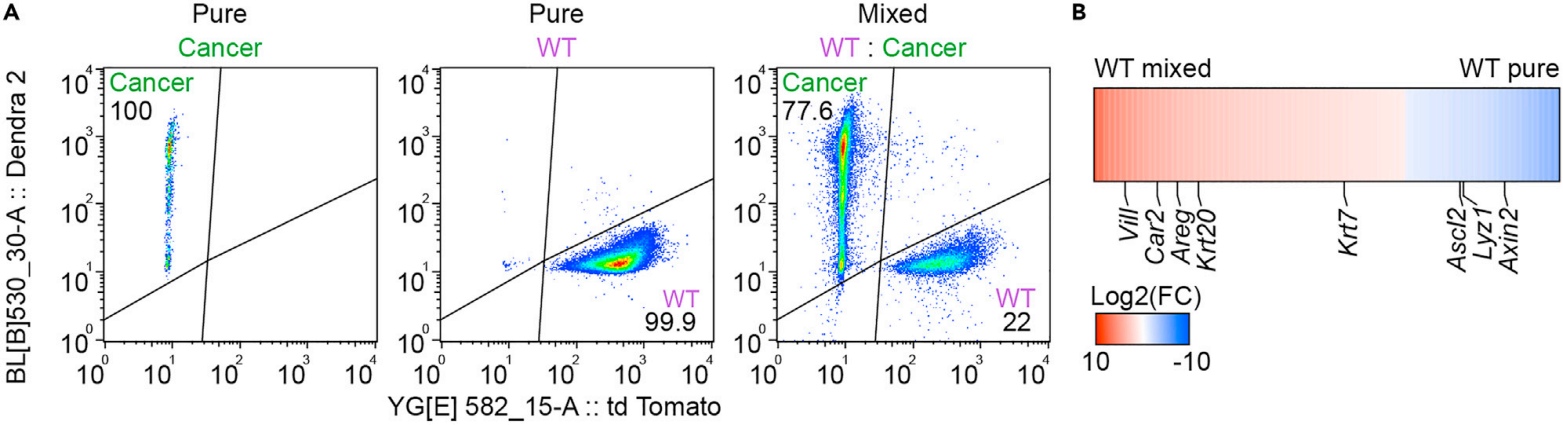
Analysis of cellular interactions using cell sorting (A) Flow cytometry sorting of wild-type and cancer cells from pure and mixed cultures, the numbers in the corners display the percentage of sorted cells. (B) Heatmap of the fold change of genes that are differentially expressed in wild-type cells upon mixing (Log2). Genes that are expressed by specific adult intestinal cell types are indicated.

These mixed cultures can be used to study cellular interactions with a variety of methods. Immuno-fluorescence staining can reveal cell competition-induced changes in cellular behavior and morphology. We have previously observed that wild-type small intestine cells revert to a fetal-like state (Krotenberg Garcia et al., 2021). This coincides which a diminished formation of crypt-like structures and reduced presence of cell types that are normally present in adult intestinal tissue, such as Paneth and intestinal stem cells (Figures 8A–8C). In addition, activation of signaling pathways can be studied using immuno-fluorescence. Wild-type cells are elimination via apoptosis and this is driven by active JNK signaling (Krotenberg Garcia et al., 2021), which can be visualized using cl-CASP3 and cJUN-pS73 antibodies (Figures 8D and 8E; Methods video S1). Specific changes in cellular behavior can be tracked by time-lapse microscopy (Figures 9A and 9B; Methods video S2 and S3). Cell sorting is used to detect changes on a population level (Figure 10A). Sorted cells can subsequently be used for further downstream analysis such as (mRNA) sequencing or proteomics. Such analysis has revealed a reversion of wild-type cells to a fetal-like state (Krotenberg Garcia et al., 2021) and reduced expression of intestinal stem cell markers (Figure 10B).

Methods video S1. 3D-reconstruction of confocal images of a mixed organoid, related to Figure 8D and steps 32–42 Cancer (green) and wild-type (magenta) cells are visualized. The organoid was stained for cJUN-pS73 (grey) and nuclei with DAPI (blue)

Methods video S2. Time-lapse series of control treated 3D reconstructed mixed intestinal organoid, related to Figure 9A and steps 43–46 Cancer (green) and wild-type (magenta) cells are visualized

Methods video S3. Time-lapse series of a competing enteroid monolayer, related to Figure 9B and steps 43–46 Cancer (green) and wild-type (magenta) cells are visualized. Arrow indicates an example of a wild-type cells that shrinks and is eliminated

## Limitations

Our protocol describes 3D mixed organoids and enteroid monolayers that are generated from adult stem cell derived cystic-growing organoid cultures. These cultures only contain epithelial cells and lack other cell types such as mesenchyme, neurons and immune cells. Therefore, the current protocol cannot be used to study the influence of a microenvironment. Extensive optimization is required to adapt this protocol for these purposes.

Currently, the maximum duration of the experiment is limited to the growth and passage requirements of the organoid cultures. The optimal duration for the mixed 3D cultures described in this protocol is three days and the maximum length is five days, by which all wild-type cells are eliminated. The mixed enteroid monolayers are viable for at least ten days, however most wild-type cells are lost within seven days after a full monolayer is formed. It is not possible to passage the organoids while preserving the mixed cellular interactions. Propagation of the long-term effects of cellular interactions can possibly be achieved by re-mixing of cells that were sorted from mixed populations.

Generation of viable mixed organoids from cultures that have incompatible basic growth conditions can be problematic. For example, when one of the essential growth factors for one of the cell populations has a negative impact on the health of another cell population. In this instance, a shared experimental medium cannot easily be designed and redefinition of the minimal medium composition is required. In addition, extreme differences in the proliferation rate of the individual cultures will impair generation of sufficient mixed organoids. Here, adaptation of the experimental design is necessary. For example, by mixing cell clumps of the slow dividing population with fast growing single cells.

## Troubleshooting

### Problem 1

Drops of BME2 attach to side of well (Step 17)

### Potential solution

Ensure unpackaged tissue culture plates are incubated in a heated CO2 incubator for at least 48 h prior to use. Choose a smaller drop size if the problem persists.

### Problem 2

(Mixed) organoids adhere to the bottom of the plate (Step 18).

### Potential solution

Invert the plate while drops are solidifying and/or use low-attachment/uncoated tissue culture plates

### Problem 3

Only a low percentage of organoids are mixed.

### Potential solution

Reduce the volume of medium that is used during aggregation (Step 13). Ensure the organoids are optimally broken (Figure 4). Increase the amount of input material.

### Problem 4

Mixed organoids fuse during the experiment.

### Potential solution

Plate less dense cultures after aggregation by adding higher volumes of medium and BME2 (Step 16, also see Figure 5).

### Problem 5

It is difficult to distinguish the two different cell populations during FACS or microscopy analysis (see “expected outcomes”).

### Potential solution

Ensure both cell populations are differentially fluorescently labelled and this is bright enough for your analysis platform of choice (see “before you begin”). In addition, a counterstain can be used in order to detect all cells in mixed organoids (e.g., DAPI or expression of a fluorescently-tagged histone H2B variant).

### Problem 6

One of the cell populations is lost before the end of the experiment (Figure 7)

### Potential solution

Adapt the input ratio during mixing (Table 1) and/or ensure cells are mixed as clumps, not single cells (Figure 4).

### Problem 7

Fluorescent signal is not equally distributed after immuno-fluorescence staining (see “expected outcomes”).

### Potential solution

Ensure BME2 drops are fully dissolved by fixation solution (Figure 6). Prolong permeabilization (Step 35) and/or increase the percentage of TX-100 in the Blocking solution.

### Problem 8

Quality of images is reduced or lost during time-lapse image acquisition (Steps 43–46).

### Potential solution

Ensure the microscope is heated and incubate the plate in the system for at least 30 min before set-up the session to avoid drift. Carefully determine maximum Z-dimension before selecting positions to ensure the organoids stay within reach during acquisition and the objective is not damaged. Ensure the system is equipped with a C02 incubator and check flow rate. Use a dry objective for prolonged time-lapse imaging to prevent loss of water or oil immersion. Alternatively, a water pump can be installed to keep a stable water immersion at the objective. Ensure, photo-toxicity and photo-bleaching are kept at a minimum level by optimization of acquisition settings (e.g., laser power, scanning time).

## Resource availability

### Lead contact

Further information and requests for resources and reagents should be directed to and will be fulfilled by the lead contact, Saskia J.E. Suijkerbuijk (s.j.e.suijkerbuijk@uu.nl).

### Materials availability

This study did not generate new unique reagents, plasmids, or organoid lines

## Data Availability

This study did not generate new unique datasets or code.
